# Frequency of cefiderocol heteroresistance among patients treated with cefiderocol for carbapenem-resistant *Acinetobacter baumannii* infections

**DOI:** 10.1093/jacamr/dlae146

**Published:** 2024-09-09

**Authors:** Ryan K Shields, Ava J Dorazio, Giusy Tiseo, Kevin M Squires, Alessandro Leonildi, Cesira Giordano, Ellen G Kline, Simona Barnini, Alina Iovleva, Marissa P Griffith, Daria Van Tyne, Yohei Doi, Marco Falcone

**Affiliations:** Department of Medicine, University of Pittsburgh, Pittsburgh, PA, USA; Center for Innovative Antimicrobial Therapy, University of Pittsburgh, Pittsburgh, PA, USA; Antibiotic Management Program, University of Pittsburgh Medical Center, Pittsburgh, PA, USA; Department of Medicine, University of Pittsburgh, Pittsburgh, PA, USA; Department of Clinical and Experimental Medicine, Azienda Osperdaliero Universitaria Pisana, University of Pisa, Pisa, Italy; Department of Medicine, University of Pittsburgh, Pittsburgh, PA, USA; Microbiology Unit, Azienda Ospedaliera Universitaria Pisana, Pisa, Italy; Microbiology Unit, Azienda Ospedaliera Universitaria Pisana, Pisa, Italy; Department of Medicine, University of Pittsburgh, Pittsburgh, PA, USA; Microbiology Unit, Azienda Ospedaliera Universitaria Pisana, Pisa, Italy; Department of Medicine, University of Pittsburgh, Pittsburgh, PA, USA; Center for Innovative Antimicrobial Therapy, University of Pittsburgh, Pittsburgh, PA, USA; Department of Medicine, University of Pittsburgh, Pittsburgh, PA, USA; Department of Medicine, University of Pittsburgh, Pittsburgh, PA, USA; Center for Innovative Antimicrobial Therapy, University of Pittsburgh, Pittsburgh, PA, USA; Center for Evolutionary Biology and Medicine, University of Pittsburgh, Pittsburgh, PA, USA; Department of Medicine, University of Pittsburgh, Pittsburgh, PA, USA; Center for Innovative Antimicrobial Therapy, University of Pittsburgh, Pittsburgh, PA, USA; Departments of Microbiology and Infectious Diseases, Fujita Health University School of Medicine, Toyoake, Aichi, Japan; Department of Clinical and Experimental Medicine, Azienda Osperdaliero Universitaria Pisana, University of Pisa, Pisa, Italy

## Abstract

**Background:**

Cefiderocol exhibits potent *in vitro* activity against carbapenem-resistant *Acinetobacter baumannii* (CRAb), but this activity has not consistently translated to improved outcomes among patients. Cefiderocol heteroresistance, or the presence of a resistant subpopulation, has been proposed as one possible explanation. The objective of this study was to explore associations between heteroresistance and outcomes of patients with CRAb infections.

**Methods:**

Baseline CRAb isolates were collected from 27 consecutive patients in the USA and Italy. Cefiderocol susceptibility was tested by broth microdilutions in triplicate. Heteroresistance was defined by population analysis profiling in duplicate. Resistance mechanisms and strain relatedness were evaluated through comparative genomic analysis.

**Results:**

Overall, 59% of infecting CRAb isolates were identified as cefiderocol-heteroresistant; rates were higher among isolates from Italy (79%) than the USA (38%). The median Charlson Comorbidity and SOFA scores were 4 and 5, respectively; 44% of patients had pneumonia, which was the most common infection type. Rates of 28-day clinical success and survival were 30% and 73%, respectively. By broth microdilution, cefiderocol MICs ≥1 mg/L were associated with higher failure rates than MICs ≤0.5 mg/L (81% versus 55%). Rates of clinical failure were numerically higher among patients infected by cefiderocol-heteroresistant compared with susceptible CRAb (81% versus 55%). Whole-genome sequencing identified a premature stop codon in the TonB*-*dependent receptor gene *piuA* in six isolates, all of which were heteroresistant.

**Conclusions:**

This pilot study supports the hypothesis that cefiderocol treatment failure may be associated with higher MICs and/or the presence of heteroresistance. Further studies are needed to confirm these findings.

## Introduction

Antimicrobial resistance presents a significant threat to patient wellbeing and a burden to healthcare infrastructure.^1^ In 2019, carbapenem-resistant *Acinetobacter baumannii* (CRAb) was projected as the fourth leading cause of death among antimicrobial resistant pathogens globally.^1^ In 2020, an estimated 7500 CRAb cases with 700 associated deaths were identified in the USA alone.^2^ Worldwide, CRAb bloodstream and respiratory tract infections are associated with excessive morbidity and mortality as evidenced by recent randomized clinical trials.^3–5^ Bloodstream infections caused by CRAb have been associated with a 16% higher attributable mortality compared with those caused by carbapenem-susceptible Gram-negative bacilli.^6^ These high rates of mortality are due, in part, to the lack of safe and effective treatment options for CRAb infections.^7^

Cefiderocol, a novel siderophore cephalosporin, demonstrates promising *in vitro* activity against CRAb;^8^ however, patients randomized to treatment with cefiderocol for CRAb infections experienced higher mortality rates when compared with best available therapy in the CREDIBLE-CR clinical trial.^5^ Beyond baseline differences between patient cohorts, one possible explanation for the observed imbalance between treatment arms is a high rate of heteroresistance to cefiderocol among the infecting CRAb isolates.^9^ Broadly, heteroresistance describes a resistant subpopulation of cells that exist within a phenotypically susceptible majority that can be enriched upon selective pressure.^10^ Evidence to support the clinical impact of antibiotic heteroresistance largely stems from vancomycin heteroresistant *Staphylococcus aureus*;^11,12^ however, observations of antibiotic heteroresistance among *A. baumannii* have been reported.^13–15^ The objective of this study was to investigate the association between cefiderocol heteroresistance and clinical outcomes of patients treated with cefiderocol at two academic institutions in Italy and the USA.

## Materials and methods

Consecutive adult patients who received cefiderocol for at least 48 hours to treat CRAb infections were included from institutions in Pisa, Italy (January 2022 to August 2022), and Pittsburgh, USA (July 2020 to May 2023). Cases were included only if the infecting CRAb isolate was identified as susceptible to cefiderocol by local susceptibility testing methods using disk diffusion or gradient strip testing at the time of treatment initiation. Infection types were defined according to the primary team and confirmed by study investigators. Patients without signs and symptoms of infection were excluded. The primary outcome of interest was clinical success at 28 days defined as a composite of survival, resolution of signs and symptoms of infection, and absence of recurrent infection or microbiologic failure following the onset of infection.^16^ Secondary outcomes included all-cause mortality and development of cefiderocol resistance following treatment. Cefiderocol resistance was determined by local susceptibility testing methods as any non-susceptible isolate according to CLSI breakpoints.^17^

Baseline isolates from patients who met the inclusion criteria were collected for further analysis. All cefiderocol minimum inhibitory concentrations (MICs) were determined in triplicate by broth microdilution using iron-depleted cation-adjusted Mueller–Hinton broth (ID-CAMHB) at a central laboratory. Cefiderocol concentrations that were tested ranged from 0.03–32 mg/L (Fetroja^®^ for injection purchased from hospital pharmacy; LOT no. 0033); MICs were interpreted according to CLSI breakpoints and were only recorded when MICs for quality control strain *Escherichia coli* ATCC 25922 were within the reference range.^17^ Monoclonal population analysis profiling (PAP) was performed to identify heteroresistance as previously described.^9,18^ In brief, a single colony was selected at random for each clinical isolate and incubated overnight in MHB. The overnight culture growth (equivalent to ≥8 log_10_ cfu/mL) was diluted and 10 µL were plated on MH agar containing cefiderocol at 2, 4, 8, 16, 32 and 64 mg/L, and a drug-free control plate. Surviving colonies were enumerated after at least 24 hours of incubation. Isolates were considered susceptible if <0.001% of colonies grew in the presence of 32 mg/L relative to colonies growing on drug-free plates (equivalent to a >5-log_10_ decrease in cfu/mL). Isolates were categorized as heteroresistant if 0.001–50% of colonies grew in the presence of 32 or 64 mg/L of cefiderocol (equal to 2× and 4× the cefiderocol resistance breakpoint). Finally, isolates were considered resistant if >50% of colonies grew in the presence of cefiderocol at 16 mg/L or greater. All isolates were tested at least twice, each time with two technical replicates to ensure reproducibility. If the categorical interpretation of heteroresistance varied between the first two tests, a third test was conducted for adjudication and mean log-kills were used for analysis. Heteroresistance was also determined by disk diffusion using CLSI methods. Isolates that yielded colonies within the zone of inhibition were considered heteroresistant; zones that were clear were measured and classified by CLSI interpretative criteria.^17^

Isolates underwent whole-genome sequencing (WGS) on the Illumina platform, and genome assembly and multilocus sequence typing were performed as described previously.^19,20^ Sequence types were determined using the Oxford typing scheme.^21^ A core genome phylogenetic tree was constructed using snippy v4.6.0 for alignment and by RAxML v8.2.12 for the tree.^22^ Antibiotic-resistance genes were identified using ResFinder.^9–11^ Protein sequences of PirA, PiuA and its orthologue PiuD were compared with those of *A. baumannii* reference strain ACICU.^23^

Statistical analyses were performed in GraphPad Prism (version 10.2.3). Categorical variables were compared by Fisher’s exact test. Continuous variables were compared using a Wilcoxon rank-sum test. Using PAP profiles, area-under-the-curve (AUC) values were determined from PAP profiles.^24^ Statistical significance was defined as a *P* < 0.05.

## Results

Isolates were collected from 27 patients with CRAb infections who were treated with cefiderocol for a median of 11 days (range, 2–50). The median age was 58 years (range, 21–87) and 44% of patients were men. Median Charlson comorbidity index, Sequential Organ Failure Assessment (SOFA) and Acute Physiology and Chronic Health Evaluation (APACHE-II) scores were 4 (range, 0–15), 5 (range, 0–15) and 20 (range, 2–31), respectively (Table 1). The primary infection sites were pneumonia (*n* = 12), skin/soft tissue infections including osteomyelitis (*n* = 6), bacteremia (*n* = 4), intra-abdominal infections (*n* = 3) and urinary tract infections (*n* = 2). Twenty-six (7/27) percent of patients received cefiderocol monotherapy; the remaining 74% (20/27) received cefiderocol in combination with at least one other antibiotic with *in vitro* activity against CRAb. Rates of 28-day clinical success and survival were 30% (8/27) and 63% (17/27), respectively. The emergence of cefiderocol resistance was documented in 26% (7/27) of patients during or following the initial treatment course.

**Table 1.** Characteristics and clinical outcomes of patients with CRAb infections treated with cefiderocol alone or in combination

Patient	Age (Sex)	Primary infection	Charlson score	SOFA	APACHE II	Treatment regimen (duration in days)	28d mortality	28d response	Emergent Resistance	Isolate	FDC MIC (mg/L)	Heteroresistance
Pitt-1	43 (F)	HAP/VAP	0	15	25	Cefiderocol (15), Ampicillin-sulbactam (8), Polymyxin B (12)	Yes	Failure	Yes	AB526	0.25	Yes
Pitt-2	71 (M)	IAI	8	8	25	Cefiderocol (37), Tigecycline (37), Ampicillin-sulbactam (8)	No	Failure	Yes	AB555	2	Yes
Pitt-3	38 (M)	HAP/VAP	3	12	22	Cefiderocol (8), Ampicillin-sulbactam (8), Inhaled colistin (8)	No	Failure	No	AB574	0.25	Yes
Pitt-4	54 (M)	HAP/VAP	4	6	23	Cefiderocol (18), Ampicillin-sulbactam (19), Polymyxin B (12)	Yes	Failure	No	AB589	1	Yes
Pitt-5	47 (M)	SSTI	3	0	2	Cefiderocol (50), Ampicillin-sulbactam (44)	No	Failure	Yes	AB648	2	Yes
Italy-1	21 (M)	BSI	0	10	20	Cefiderocol (19), Tigecycline (11)	No	Failure	Yes	E0396	4	Yes
Italy-2	61 (F)	IAI	15	11	28	Cefiderocol (8), Fosfomycin (11)	No	Failure	Yes	E0418	1	Yes
Italy-3	71 (F)	HAP/VAP	6	11	31	Cefiderocol (3)	Yes	Failure	No	E0420	1	Yes
Italy-4	87 (F)	BSI	5	4	11	Cefiderocol (13)	Yes	Failure	No	E0422	1	Yes
Italy-5	53 (F)	SSTI	2	7	15	Cefiderocol (11), Tigecycline (16)	No	Success	No	E0423	0.5	Yes
Italy-6	85 (F)	SSTI	8	14	30	Cefiderocol (4)	Yes	Failure	No	E0424	8	Yes
Italy-7	80 (F)	HAP/VAP	5	2	8	Cefiderocol (10), Tigecycline (6)	No	Failure	Yes	E0425	2	Yes
Italy-8	80 (F)	UTI	7	4	15	Cefiderocol (11), Ampicillin-sulbactam (11)	Yes	Failure	No	E0427	1	Yes
Italy-9	72 (F)	HAP/VAP	6	5	24	Cefiderocol (8)	No	Success	No	E0428	0.5	Yes
Italy-10	44 (F)	BSI	0	12	27	Cefiderocol (2), Tigecycline (2)	Yes	Failure	No	E0431	2	Yes
Italy-11	25 (M)	HAP/VAP	1	2	7	Cefiderocol (10), Tigecycline (10)	No	Success	No	E0432	4	Yes
Pitt-6	58 (M)	HAP/VAP	3	9	25	Cefiderocol (12), Polymyxin B (14), Minocycline (10), Inhaled colistin (14)	No	Success	No	AB536	1	No
Pitt-7	48 (M)	HAP/VAP	2	1	7	Cefiderocol (15), Minocycline (15)	No	Success	No	AB545	1	No
Pitt-8	60 (M)	IAI	5	15	20	Cefiderocol (6), Tigecycline (24), Ampicillin-sulbactam (24)	Yes	Failure	No	AB570	1	No
Pitt-9	62 (F)	SSTI	5	4	14	Cefiderocol (6), Minocycline (27)	Yes	Failure	No	AB586	2	No
Pitt-10	70 (M)	HAP/VAP	10	11	27	Cefiderocol (4), Tigecycline (4)	Yes	Failure	No	AB603	0.5	No
Pitt-11	47 (F)	HAP/VAP	1	5	21	Cefiderocol (25), Minocycline (23), Polymyxin B (18)	No	Failure	No	AB605	0.5	No
Pitt-12	79 (M)	SSTI	7	0	9	Cefiderocol (43)	No	Success	No	AB616	0.5	No
Pitt-13	63 (F)	BSI	3	2	6	Cefiderocol (31)	No	Success	No	E0368	0.12	No
Italy-12	44 (M)	SSTI	7	4	8	Cefiderocol (39), Tigecycline (42)	No	Failure	Yes	E0421	0.5	No
Italy-13	33 (F)	HAP/VAP	0	4	8	Cefiderocol (7), Tigecycline (7)	No	Failure	No	E0429	0.5	No
Italy-14	31 (F)	UTI	2	5	5	Cefiderocol (11)	No	Success	No	E0430	0.25	No

APACHE II, acute physiology and chronic health evaluation II; BSI, bloodstream infection; F, female; FDC, cefiderocol; HAP, hospital-associated pneumonia; IAI, Intra-abdominal infection; M, male; MIC, minimum inhibitory concentration; SOFA, sequential organ failure assessment; SSTI, skin and soft tissue infection; UTI, urinary tract infection; VAP, ventilator-associated pneumonia.

Confirmatory broth microdilution and disk diffusion testing were performed on baseline isolates. By broth microdilution testing, the median cefiderocol MIC was 1 mg/L (range, 0.12–8 mg/L) and 96% (26/27) of isolates were categorized as susceptible using the CLSI a breakpoint of ≤4 mg/L. By comparison only 48% (13/27) of isolates were classified as susceptible by disk diffusion testing. For isolates classified as non-susceptible by disk diffusion, colonies were identified within the zone of inhibition for 79% [11/14; Figure S1 (available as Supplementary data at *JAC-AMR* Online)]. Heteroresistance defined by PAP was detected in 59% (16/27) of isolates. Among heteroresistant isolates, the proportion of colonies growing at 32 mg/L or 64 mg/L relative to drug-free control ranged from 0.0016%–0.165% and 0.0017%–0.2846%, respectively (Table S1 and Figure S2). The mean AUC for heteroresistant isolates was significantly higher than for susceptible isolates (260.1 versus 163.0; *P *< 0.001). Heteroresistance was numerically more common among isolates with cefiderocol MICs ≥1 mg/L [75% (12/16)] compared with isolates with MICs ≤0.5 mg/L [36% (4/11); *P = *0.06, Figure S3].

Clinical success was achieved in 45% (5/11) of patients treated with cefiderocol-susceptible isolates (as determined by PAP) compared with 19% (3/16) for those infected with cefiderocol-heteroresistant isolates. When stratified by MIC, success was achieved in 45% (5/11) of patients infected with isolates demonstrating a cefiderocol MIC ≤0.5 mg/L compared with 19% (3/16) of patients infected with isolates demonstrating a cefiderocol MIC ≥1 mg/L (Figure S4). Rates of 28-day mortality did not vary significantly by the presence or the absence of heteroresistance (44% versus 27%). Subsequent development of cefiderocol resistance was identified in 38% (6/16) of patients infected by heteroresistant isolates compared with 9% (1/11) of susceptible isolates.

**Figure 1.**
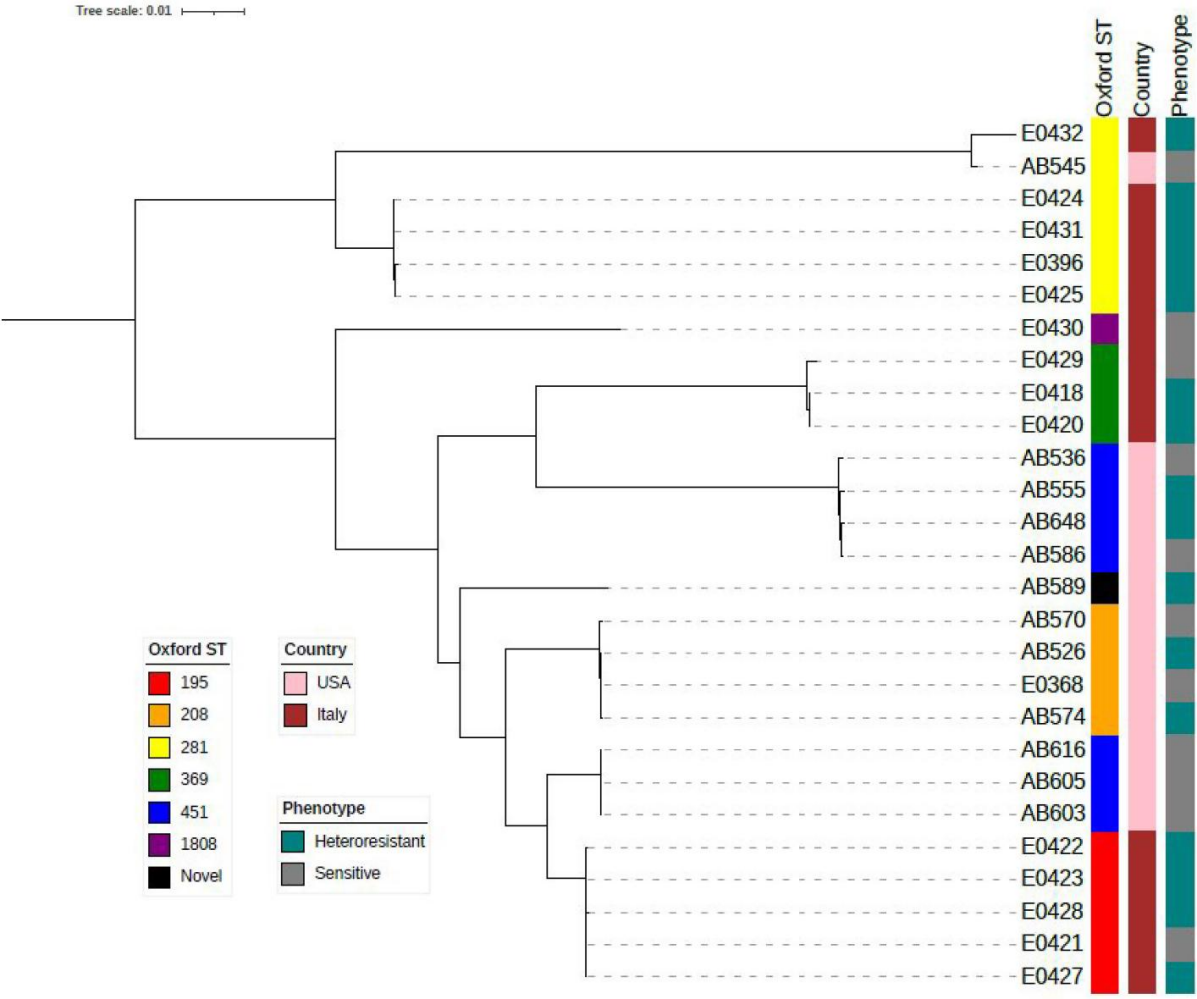
Phylogenetic tree and characterization of CRAb isolates from Pisa, Italy and Pittsburgh, USA.

Whole-genome sequence analysis identified clonal clusters unique to each centre (Figure 1); yet, isolates were genetically diverse and represented seven unique sequence types (ST). The most common Oxford STs were ST281 (*n* = 6) and ST451 (*n* = 7). Rates of heteroresistance did not vary by ST or phylogenetic cluster; however, 79% (11/14) of isolates from Italy were classified as heteroresistant compared with 38% (5/13) of isolates from the USA (*P = *0.05). Loss-of-function mutations in genes encoding TonB-dependent receptors (TBDR; *pirA* and *piuA*) were detected in 26% (7/27) of isolates. Eighty-six percent (6/7) of isolates with mutations in TBDR genes displayed a heteroresistant phenotype, including all six isolates with mutations encoding a premature stop codon in *piuA* (Table S2).

## Discussion

Heteroresistance is a poorly understood form of antibiotic resistance that may or may not be linked to poor clinical outcomes for patients.^25^ Unlike classic resistance, heteroresistance is not often detected by standard antibiotic susceptibility testing, and therefore represents a potentially underappreciated risk factor for treatment failure. Cefiderocol heteroresistance has now been documented in several reports,^9,18,24,26–28^ and is hypothesized to explain higher than anticipated rates of treatment failure for patients with CRAb infections.^9^ Against CRAb, reported rates of cefiderocol heteroresistance range from 47% to 59%,^9,18^ which is in agreement with the rate of 59% in the present study. Interestingly, we found that rates of heteroresistance varied from 79% of isolates collected in Italy to 38% of isolates collected at a single center in the USA. This variability could not be explained by a specific clonal cluster or mutations in specific antibiotic resistance-associated genes, suggesting that regional variability may be a key factor in the prevalence heteroresistance. Regional variability has also been described for cefiderocol heteroresistance against *Pseudomonas aeruginosa.*^24^ It is also possible that varying treatment approaches or clinical factors at each center contributes to the rate of heteroresistance.

Although experimental approaches and definitions used to identify cefiderocol heteroresistance vary across studies, it is clear that this is a common phenomenon among CRAb collected from infected patients. Herein, we attempted to associate the presence of cefiderocol heteroresistance with outcomes of patients treated with cefiderocol. In doing so, we found that rates of treatment failure were numerically higher among patients infected by CRAb isolates characterized as heteroresistant compared with patients infected by susceptible isolates according to PAP (81% versus 55%, respectively). Both heteroresistance and clinical failures were numerically more common among isolates with cefiderocol MICs ≥1 mg/L. While notable, these data should be interpreted cautiously given the small number of patients included in this study, a wide range of infectious syndromes evaluated and routine use of cefiderocol combination therapy. In addition, durations of cefiderocol exposure varied across patients from 2 to 50 days. Heteroresistant subpopulations are typically identified in the face of ongoing selective pressure and it is unclear if short exposures or the presence of heteroresistant subpopulations at baseline portend worse outcomes.^9^ Prolonged clinical exposures, on the other hand, have been reported in emergent heteroresistance during cefiderocol treatment for a complicated *P. aeruginosa* infection.^26^

The available data also suggest that heteroresistance could be an initial step in the evolution of cefiderocol resistance.^29^ In our experience, the emergence of cefiderocol resistance was identified in 38% of isolates classified as heteroresistant compared with 9% of susceptible isolates. The development of cefiderocol resistance against CRAb is mediated by multiple molecular mechanisms, including mutations in *Acinetobacter-*derived cephalosporinase, PBP3, and TBDR genes *piuA* and *pirA.*^19,30,31^ We identified pre-existing TBDR gene mutations in 26% of isolates prior to treatment with cefiderocol, among which 86% were classified as heteroresistant by PAP. It is plausible that TBDR mutations specifically confer an initial step towards heteroresistance, or more concerningly, outright resistance following treatment. Our findings corroborate a prior report linking mutations in TBDR genes to cefiderocol heteroresistance against *P. aeruginosa.*^24^ While the cumulative data of these two studies are limited to a relatively small number of isolates, the findings underscore a potentially important predictor of cefiderocol resistance. Moreover, the data highlight a disconnect between high rates of cefiderocol *in vitro* activity,^8^ and a growing number of reports describing clinical failures and/or emergence of resistance against CRAb.^19,32–34^ This disconnect is perpetuated by the technical challenges that have been described in testing cefiderocol susceptibility against *A. baumannii,*^35^ which may preclude MIC-based clinical decision making. Consistent with our findings, high error rates have been reported for cefiderocol disk diffusion testing against *A. baumannii* specifically.^36^ In fact, heteroresistance may contribute to this discordance when compared with the gold standard broth microdilution testing.^37^

Our findings contrast those recently reported from a post-hoc evaluation of isolates collected from patients enrolled in the CREDIBLE-CR trial, which did not identify associations between heteroresistance and clinical cure, microbiologic eradication, or mortality.^18^ In a post-hoc analysis, 38 CRAb isolates were available from patients treated with cefiderocol. By broth microdilution testing, 5% (2/38) were classified as resistant. Among the remaining 36 isolates, 19.4%, 50% and 30.6% were classified as susceptible, heteroresistant or resistant by PAP. Excluding a single patient with a complicated urinary tract infection, 100% (7/7) of patients infected by isolates classified as susceptible to cefiderocol by PAP died by the test-of-cure visit. Corresponding rates of death for those infected by cefiderocol-heteroresistant or resistant isolates were 22% (4/18) and 75% (8/12), respectively. It is worth noting that a significant proportion of patients in this trial are believed to have deteriorated due to underlying conditions and septic shock that was present prior to receipt of cefiderocol.^5^ Such clinical factors may mask potential associations between cefiderocol MIC, drug exposures, or the presence of heteroresistance and outcomes. Nonetheless, it is unclear why failure rates were numerically highest among isolates classified as susceptible to cefiderocol.

Finally, it is important to acknowledge the limitations of the current analysis. First, our study was limited by a small number of patients, its retrospective, observational nature and use of cefiderocol to treat various infection types. As such, we view these data as hypothesis-generating. Future, multicentre studies would be needed to identify definitive associations between cefiderocol heteroresistance, MICs and clinical outcomes of patients infected by CRAb. Secondly, we recognize that defining heteroresistance is subject to methodological variation and definitions applied. Here, we identified heteroresistance as a ≤5-log_10_ cfu/mL killing at a cefiderocol concentration of ≥32 mg/L, which is equal to 4× the non-susceptibility breakpoint for cefiderocol (MIC ≥8 mg/L). To account for potential differences in our definition compared with those that have been published previously,^9,24^ we derived AUC measurements from PAP studies. These analyses demonstrated clear differences between heteroresistant and susceptible isolates (Table S1). Lastly, we did not investigate mechanisms of cefiderocol heteroresistance beyond WGS analysis. Heteroresistance has been previously reported to be unstable and potentially overcome by partnering cefiderocol with β-lactamase inhibitors like avibactam.^38^ Consistent with current recommendations, cefiderocol may be best utilized in combination with other *in vitro* active antibiotics for treatment of CRAb infections.^7,39^ Despite these limitations, we have identified potential associations between elevated cefiderocol MICs and the presence of heteroresistance, both of which may be associated with higher rates of treatment failure for patients with CRAb infections.

## Supplementary Material

dlae146_Supplementary_Data

## Supplementary data

Figures S1 to S4 and Tables S1 and S2 are available as Supplementary data at *JAC-AMR* Online.
