# Supplementary material for: Frequency of cefiderocol heteroresistance among patients treated with cefiderocol for carbapenem-resistant Acinetobacter baumannii infections
Source: JAC Antimicrob Resist. 2024 Sep 9;6(5):dlae146. doi: 10.1093/jacamr/dlae146 (PMC11382143; doi:10.1093/jacamr/dlae146)
Supplement: dlae146_Supplementary_Data [file dlae146_supplementary_data.pdf]

Supplementary Figure 1. Heteroresistance identified by cefiderocol disk diffusion testing of carbapenem-resistant *Acinetobacter baumannii* clinical isolates.

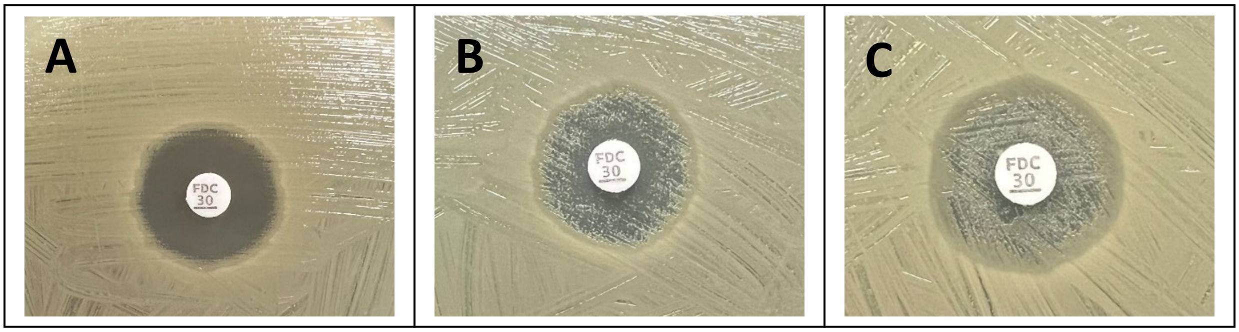

Note. Panel A demonstrates an isolate classified as susceptible by disk diffusion testing. Panels B and C demonstrate isolates classified as heteroresistant based on the presence of colonies within the zone of inhibition.

Supplementary Figure 2. Mean survival values for each isolate across doubling dilutions of cefiderocol distinguished by classification as heteroresistant or susceptible

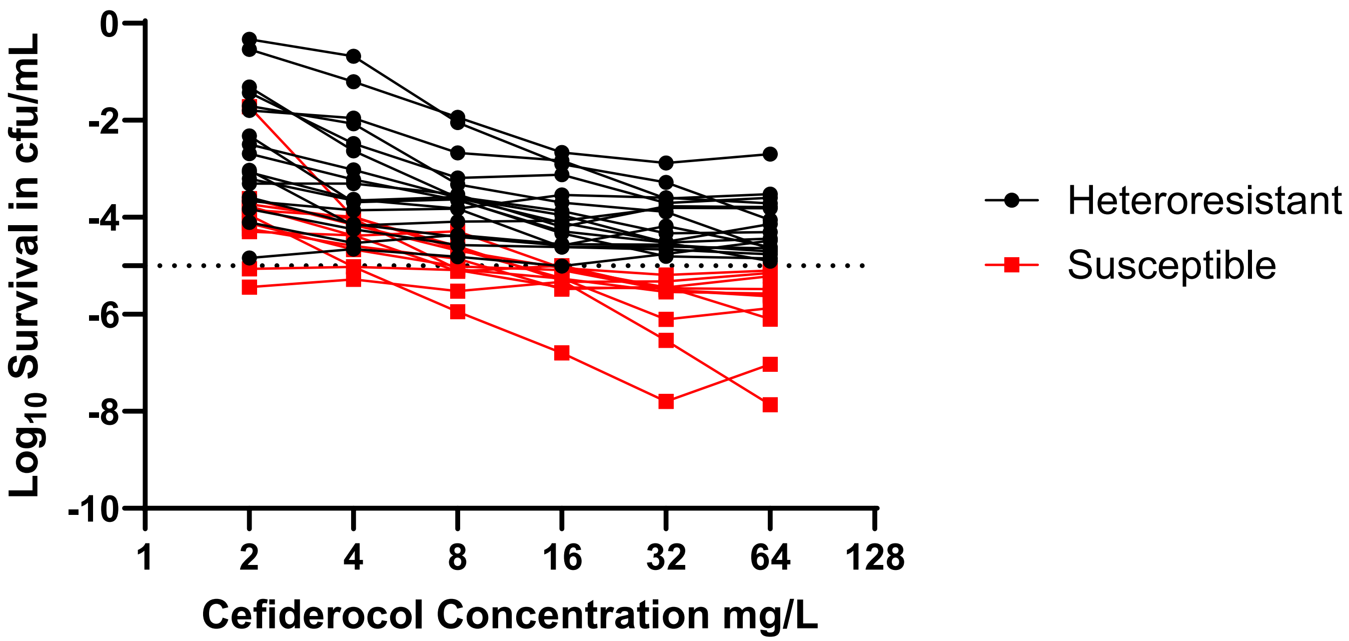

Note. Horizontal dotted line at -5log$_{10}$ signifies the predefined break between heteroresistant and susceptible (<0.001% of isolates growing at 32mg/L, equivalent to -5log$_{10}$).

Supplementary Figure 3. Distribution of isolates by cefiderocol MICs by PAP susceptibility classification.

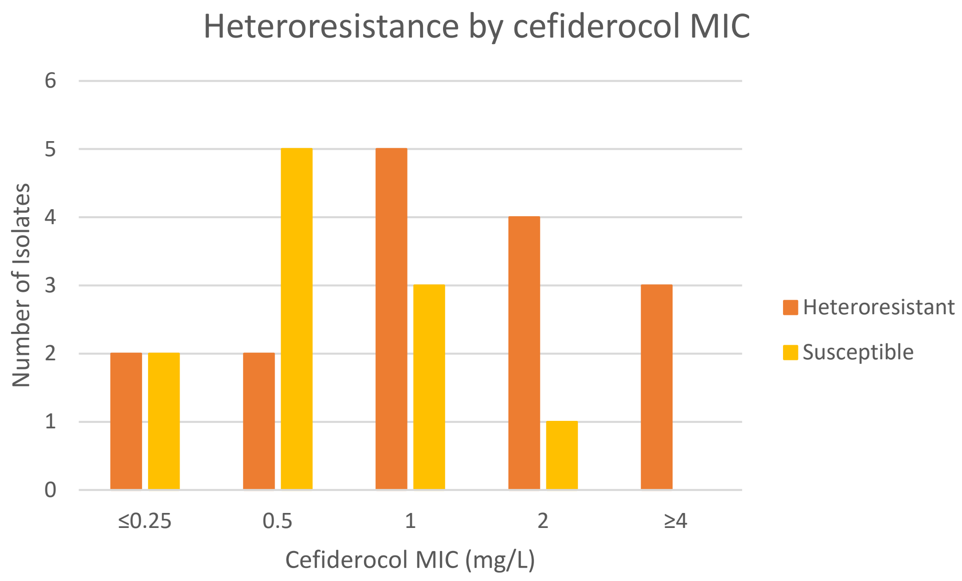

Abbreviations: MIC = minimum inhibitory concentration; PAP = population analysis profile

Supplementary Figure 4. Distribution of patients experiencing clinical success or failure separated by baseline isolate cefiderocol MIC.

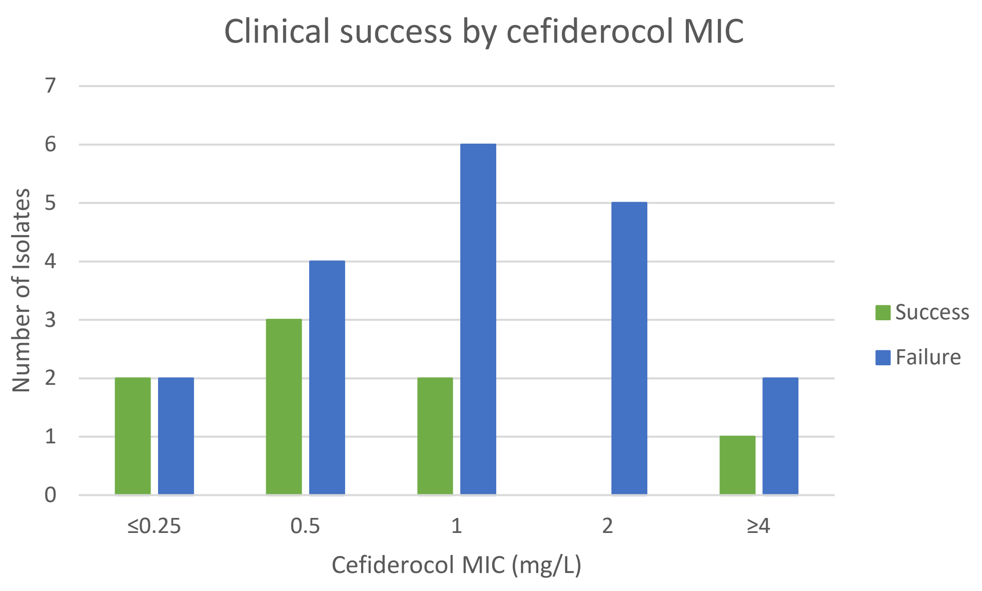

Abbreviation: MIC = minimum inhibitory concentration

Supplementary Table 1. Summary of PAP results for each baseline isolate.

Patient	Isolate	FDC MIC (µg/mL)	HR classification	Mean log-kill at 32µg/mL	% reduction[1]	Mean log-kill at 64µg/mL	% reduction[1]	AUC
**Italy-3**	E0420	1	HR	-2.85	0.1650%	-2.69	0.2846%	351.5
**Italy-6**	E0424	8	HR	-3.62	0.0355%	-3.52	0.0520%	309.5
**Italy-2**	E0418	1	HR	-3.70	0.0394%	-3.60	0.0727%	298.7
**Italy-11**	E0432	4	HR	-3.59	0.0269%	-3.71	0.0200%	284
**Italy-7**	E0425	2	HR	-3.77	0.0183%	-3.79	0.0200%	272.9
**Italy-8**	E0427	1	HR	-3.81	0.0185%	-3.81	0.0218%	267.7
**Italy-5**	E0423	0.5	HR	-4.51	0.0044%	-4.14	0.0239%	252.7
**Pitt-5**	AB648	2	HR	-4.33	0.0049%	-4.30	0.0053%	253.9
**Pitt-4**	AB589	1	HR	-4.52	0.0051%	-4.44	0.0053%	244.1
**Pitt-1**	AB526	0.25	HR	-4.76	0.0019%	-4.48	0.0040%	213.5
**Italy-9**	E0428	0.5	HR	-4.18	0.0067%	-4.61	0.0038%	236.8
**Pitt-2**	AB555	2	HR	-4.60	0.0028%	-4.64	0.0023%	230.2
**Italy-1**	E0396	4	HR	-3.88	0.0140%	-4.66	0.0030%	270.4
**Pitt-3**	AB574	0.25	HR	-4.56	0.0032%	-4.70	0.0022%	224.7
**Italy-10**	E0431	2	HR	-4.66	0.0030%	-4.76	0.0028%	220.8
**Italy-4**	E0422	1	HR	-4.80	0.0016%	-4.84	0.0017%	229.8
**Pitt-7**	AB545	1	S	-5.19	0.0008%	-5.09	0.0010%	197.7
**Pitt-13**	E0368	0.12	S	-5.32	0.0005%	-5.15	0.0008%	178.2
**Pitt-8**	AB570	1	S	-5.44	0.0004%	-5.22	0.0007%	190.9
**Pitt-10**	AB603	0.5	S	-5.47	0.0004%	-5.48	0.0003%	174.4
**Pitt-12**	AB616	0.5	S	-5.54	0.0004%	-5.57	0.0004%	176.8
**Pitt-11**	AB605	0.5	S	-5.50	0.0006%	-5.60	0.0005%	178.1
**Italy-13**	E0429	0.5	S	-5.50	0.0004%	-5.63	0.0003%	176.7
**Italy-14**	E0430	0.25	S	-6.11	0.0003%	-5.87	0.0002%	161
**Italy-12**	E0421	0.5	S	-5.42	0.0006%	-6.09	0.0001%	167.1
**Pitt-6**	AB536	1	S	-7.79	0.0000%	-7.03	0.0001%	70
**Pitt-9**	AB586	2	S	-6.54	0.0002%	-7.87	0.0000%	122.5

Abbreviations: AUC = area under the curve; FDC = cefiderocol; HR = heteroresistant; S = susceptible

[1] The percent reduction was calculated as the ratio of colonies growing on drug-free agar relative to cefiderocol-containing agar.

Supplementary Table 2. Molecular characteristics of isolates classified as cefiderocol-heteroresistant and susceptible.

Patient	Isolate	HR by PAP	FDC MIC (mg/L)	Oxford ST	Acquired bla_{OXA}	Intrinsic bla_{OXA}	bla_{ADC}	PBP3	*piuA*	*pirA*	Other antibiotic resistance genes detected
Italy-4	E0422	HR	1	195	OXA-23	OXA-66	ADC-73	A515V	Intact	Intact	*strA, strB, aphA1, armA,* bla_{TEM-1D}, *mph*(E), *msr*(E), *sul2, tet*(B)
Italy-5	E0423	HR	0.5	195	OXA-23	OXA-66	ADC-73	A515V	Intact	Intact	*strA, strB, aphA1, armA,* bla_{TEM-1D}, *mph*(E), *msr*(E), *sul2, tet*(B)
Italy-8	E0427	HR	1	195	OXA-23	OXA-66	ADC-73	A515V	Intact	Intact	*strA, strB, aphA1, armA,* bla_{TEM-1D}, *mph*(E), *msr*(E), *sul2, tet*(B)
Italy-9	E0428	HR	0.5	195	OXA-23	OXA-66	ADC-73	A515V	Intact	Intact	*strA, strB, aphA1, armA,* bla_{TEM-1D}, *mph*(E), *msr*(E), *sul2, tet*(B)
Pitt-1	AB526	HR	0.25	208	OXA-23	OXA-80	ADC-30	WT	Intact	Intact	*strA, strB,* bla_{TEM-1D}, *sul2, tet*(B)
Pitt-3	AB574	HR	0.25	208	OXA-23	OXA-80	ADC-30	WT	Intact	Intact	*strA, strB, aphA1,* bla_{TEM-1D}, *catB88, sul2, tet(B)*
Italy-1	E0396	HR	4	281	Disrupted	OXA-66	ADC-30	K235N	T385N; D386*	Intact	*aadA1, strA, strB, aphA1, armA, catB8, mph*(E), *msr*(E), *sul1, tet*(B)
Italy-6	E0424	HR	8	281	OXA-23 (P225T)	OXA-66	ADC-30	K235N	T385N; D386*	Intact	*aadA1, strA, strB, aphA1, armA, catB8, mph*(E), *msr*(E), *sul1, tet*(B)
Italy-7	E0425	HR	2	281	OXA-23	OXA-66	ADC-30	K235N	T385N; D386*	Intact	*aadA1, strA, strB, aphA1, armA, catB8, mph*(E), *msr*(E), *sul1, tet*(B)
Italy-10	E0431	HR	2	281	OXA-23	OXA-66	ADC-30	K235N	T385N; D386*	Intact	*aadA1, strA, strB, aphA1, armA, catB8, mph*(E), *msr*(E), *sul1, tet*(B)
Italy-11	E0432	HR	4	281	OXA-23	OXA-82	ADC-33	WT	Intact	Intact	*aphA6, sul1, aadA2, aadB*
Italy-2	E0418	HR	1	369	OXA-23	OXA-66	ADC-73	N392T	Q217*	Intact	*aadA1, strA, strB, aphA1, armA, catB8, mph*(E), *msr*(E), *sul1, tet*(B)
Italy-3	E0420	HR	1	369	OXA-23	OXA-66	ADC-73	N392T	Q217*	Intact	*aadA1, strA, strB, aphA1, armA, catB8, mph*(E), *msr*(E), *sul1, tet*(B)
Pitt-2	AB555	HR	2	451	OXA-23	OXA-66 (delA at 325, fs)	ADC-56	T526S	Intact	Intact	*aadA1, strA, strB, armA, catB8, mph*(E), *msr*(E), *sul1, sul2, tet*(B)
Pitt-5	AB648	HR	2	451	OXA-23	OXA-66 (delA at 325, fs)	ADC-73 (R148Q)	WT	Intact	Intact	*strA, aphA1, strB, mph*(E), *msr*(E), *sul2, tet*(B)
Pitt-4	AB589	HR	1	new ST	Missing	OXA-113	ADC-30	WT	Intact	Intact	*strA, strB, mph*(E), *msr*(E), *tet*(B)
Italy-12	E0421	S	0.5	195	OXA-23	OXA-66	ADC-73	A515V	Intact	Intact	*strA, strB, aphA1, armA,* bla_{TEM-1D}, *mph*(E), *msr*(E), *sul2, tet*(B)
Pitt-8	AB570	S	1	208	OXA-23	OXA-66	ADC-162	WT	Intact	Y650*	*aadA1, strA, aphA1, aphA6, strB, armA,* bla_{TEM-1D}, *catB8, mph*(E), *msr*(E), *sul1, sul2, tet*(B)
Pitt-13	E0368	S	0.12	208	OXA-23	OXA-80	ADC-30	WT	Intact	Intact	*strA, strB, aphA6,* bla_{TEM-1D}, *sul2, tet*(B)
Pitt-7	AB545	S	1	281	OXA-23	OXA-82	ADC-33	WT	Intact	Intact	None found
Italy-13	E0429	S	0.5	369	OXA-23	OXA-66	ADC-73	N392T	Intact	Intact	*aadA1, strA, strB, armA, catB8, mph*(E), *msr*(E), *sul1, tet*(B)
Pitt-6	AB536	S	1	451	OXA-23	OXA-66 (delA at 325, fs)	ADC-30	N392T	Intact	Intact	*strA, strB, armA, mph*(E), *msr*(E), *sul1, sul2, arr-2, cmlA1*
Pitt-9	AB586	S	2	451	OXA-23	OXA-66 (delA at 325, fs)	ADC-56	WT	Intact	Intact	*strA, strB, armA, mph*(E), *msr*(E), *sul2, tet*(B)
Pitt-10	AB603	S	0.5	451	OXA-23	OXA-66	ADC-73	A515V	Intact	Intact	*strA, strB, aphA1, armA,* bla_{TEM-1D}, *mph*(E), *msr*(E), *tet*(B)
Pitt-11	AB605	S	0.5	451	OXA-23	OXA-66	ADC-73	A515V	Intact	Intact	*strA, strB, aphA1, armA,* bla_{TEM-1D}, *mph*(E), *msr*(E), *tet*(B)
Pitt-12	AB616	S	0.5	451	OXA-23	OXA-66	ADC-73	A515V	Intact	Intact	*strA, strB, aphA1, armA,* bla_{TEM-1D}, *mph*(E), *msr*(E), *tet*(B)
Italy-14	E0430	S	0.25	new ST	OXA-72	OXA-66	ADC-30	WT	Intact	Intact	*strA, strB, armA, mph*(E), *msr*(E), *sul1, sul2, tet*(B), *aac(6')-1p*

Note. All isolates were classified as Pasteur sequence type 2

* stop codon

Abbreviations: FDC = Cefiderocol; FS = frameshift; HR = heteroresistant; MIC = minimum inhibitory concentration; PAP = population analysis profile; S = susceptible; ST = sequence type; WT = wild-type
